# Supplementary material for: Knowledge, Attitude and Practices towards the Tiger Mosquito Aedes Albopictus. A Questionnaire Based Survey in Lazio Region (Italy) before the 2017 Chikungunya Outbreak
Source: Int J Environ Res Public Health. 2020 Jun 3;17(11):3960. doi: 10.3390/ijerph17113960 (PMC7312532; doi:10.3390/ijerph17113960)
Supplement: Supplementary file 1 [file ijerph-17-03960-s001.pdf]

Supplementary Table 1. Frequency of answers to knowledge, attitude and practice questions in the three groups. For question with multiple answers (*i.e.* knowledge of *Aedes* cycle), we reported the frequency of each answer.

domain	question	Value	Malayalis (N=204)	Punjabis (N=266)	Italians (N=1109)
knowledge	Where tiger mosquitoes lay eggs and larvae develop?	Don’t know	0.17	0.26	0.08
		Bare ground, vegetation	0.04	0.12	0.66
		Walls	0.05	0.13	0.24
		small water containers, storm drains	0.76	0.47	0.89
	What is the preferred biting time of tiger mosquitoes?	don't know	0.20	0.07	0.20
		Early morning or late afternoon	0.34	0.29	0.31
		night	0.46	0.65	0.49
	What diseases may be transmitted by tiger mosquitoes, if any?	None	0.22	0.33	0.45
		Can transmit diseases but don’t know which one	0.50	0.15	0.49
		dengue, chikungunya or yellow fever	0.24	0.09	0.01
		malaria	0.04	0.43	0.05
attitude	How often are you worried by diseases that Aedes mosquitoes may transmit?	Never / rarely	0.51	0.23	0.67
		Sometimes	0.20	0.50	0.17
		very worried / extreme worried	0.29	0.27	0.16
	How much do you feel disturbed by tiger mosquitoes bites?	Not at all	0.09	0.06	0.05
		Neutral/somehow	0.90	0.80	0.37
		A lot/ extremely	0.01	0.14	0.58
	How often do you feel disturbed at home by tiger mosquitoes?	Never / rarely	0.32	0.62	0.28
		Sometimes	0.35	0.24	0.26
		Very often / always	0.33	0.14	0.46
practice	what do you do to avoid Aedes bites?	Nothing	0.58	0.34	0.53

		Environmental repellents at home	0.23	0.04	0.19
		personal repellents	0.15	0.4	0.28
		Other	0.05	0.23	0.01
